# Hair follicle bulge cultures yield class III β-tubulin-positive melanoglial cells

**DOI:** 10.1007/s00418-015-1312-8

**Published:** 2015-02-28

**Authors:** H. Locher, N. Saadah, S. de Groot, J. C. M. J. de Groot, J. H. M. Frijns, M. A. Huisman

**Affiliations:** Department of Otorhinolaryngology and Head and Neck Surgery, Leiden University Medical Center, Albinusdreef 2, 2333 ZA Leiden, The Netherlands

**Keywords:** Hair follicle, Neural crest, Stem cell, TUBB3, Melanocyte, Glia

## Abstract

Class III β-tubulin (TUBB3)-positive cells from the hair follicle bulge are thought to be neuronal cells derived from a local neural crest stem cell. However, TUBB3 has recently been shown to be expressed in the melanocytic lineage. To evaluate the neural-crest-associated immunophenotype of TUBB3-positive cells from hair follicle bulge explants, we dissected hair follicle bulges out from mouse whisker pads and cultured for 1 month and assessed outgrowing cells by means of immunocytochemistry using the biomarkers TUBB3, nestin, NGFR, SOX9, TYRP1 and laminin. Large amounts of TUBB3-positive cells could be cultured that co-expressed nestin, NGFR, SOX9 and, to a lesser degree, TYRP1, matching a melanoglial phenotype. In addition, a small population of TUBB3-negative but laminin-positive cells was found, which presumably are of glial origin. It can be concluded that cells of melanoglial origin can easily be obtained from hair follicle bulge explants. These cells may be of use in experimental animal or human disease and wound healing models. Notably, the TUBB3-positive cells are of melanoglial rather than neuronal origin.

## Introduction

Stem cells that are harvested from adult human tissues are considered the gold standard for autologous cell-based therapies (Prasongchean and Ferretti 2012). Adult human neural-crest-derived stem cells (NCSCs) are promising candidates for the use in regenerative medicine; niches have been described in the dorsal root ganglia, gut epithelium, cornea epithelium, heart muscle, inferior turbinate, dental and periodontal tissue and the hair follicle bulge (Kaltschmidt et al. 2012). The advantage of hair follicle bulge stem cells (HFBSCs) is that they can be harvested in a minimally invasive way and that they can be isolated without proteolysis.

Nestin-positive cells from the hair follicle bulge area have been reported to be multipotent stem cells and able to differentiate into various neural-crest-derived lineages (Sieber-Blum et al. 2004; Amoh et al. 2012). It has been claimed that these nestin-positive cells differentiate into neuronal cells specifically expressing TUBB3, a widely used neural biomarker.

However, it has recently been demonstrated that mouse and human melanocytes also express TUBB3 (Akasaka et al. 2009; Adameyko et al. 2012; Locher et al. 2013). As melanocytes, similar to peripheral neurons, derive from the neural crest, we wondered whether the TUBB3-positive cells cultured from hair follicle bulge explants could be such melanocytes. If so, we should be extremely reticent about TUBB3-positive cells cultured from hair follicle bulge explants, presumed to be committed toward the neuronal lineage.

To address this issue, we investigated the immunophenotype of the cells migrating out of mouse hair follicle bulge explants by immunostaining with biomarkers specific for the neural crest and glial and melanocytic lineages.

## Materials and methods

### Specimens

Hair follicles were dissected from the whisker pads of surplus C57BL/6 mice (*n* = 7; 126 hair follicles), which were obtained from the LUMC central Animal Facility. Their use was approved by the LUMC Animal Experiments Committee (DEC permit 10172).

### Cell cultures

Hair follicle bulges were isolated from mouse whisker pads according to a protocol previously described by Sieber-Blum et al. (2004). In summary, hair follicles were dissected out from the whisker pads, the follicle was transected below and above the bulge region, and a longitudinal incision was made in the capsule. The bulge was rolled out of the capsule, and explants were transferred to 6-well cell culture plates pre-coated with collagen l (Sigma-Aldrich) and allowed to attach for 1 h at 37 °C in a humidified incubator with 5 % CO_2_ prior to addition of culture medium. Culture medium consisted of alpha MEM (Bio-Whittaker) containing 5 % chicken embryo extract (Seralab), 10 % fetal bovine serum (Life Technologies), 1 % GlutaMAX (Life Technologies) and 1 % antibiotic/antimycotic solution (Sigma-Aldrich). Medium was refreshed every other day. To allow as many cells as possible to migrate out of the explant, cultures were maintained for 4 weeks followed by fixation in 1 % formaldehyde in phosphate-buffered saline for 30 min and immunocytochemical analysis. Cell lines RT4D6P2T (schwannoma cells; ATCC) and melan-Ink4a2 (melanocytes; Wellcome Trust Functional Genomics Cell Bank) were used as positive controls and were cultured according to manufacturer’s instructions.

### Immunocytochemistry

Immunocytochemical protocols were similar to the ones described previously (Locher et al. 2013). Primary antibodies used in this study were rabbit anti-laminin (1:200, Z009701, Dako), mouse anti-nestin (1:200, Biosensis, M-1385-100), rabbit anti-SOX9 (1:500, Millipore, AB5535), rabbit anti-NGFR (1:200, Millipore, 07-476), mouse anti-TUBB3 (1:200, Abcam, ab78078) and rabbit anti-TYRP1 (1:50, Santa Cruz Biotechnology, sc-25543). Secondary antibodies were Alexa Fluor 488 goat anti-rabbit (1:500, Invitrogen, A-11034) and Alexa Fluor 555 goat anti-mouse (1:500, Invitrogen, A-21422). To confirm secondary antibody specificity, negative controls were included in which the primary antibodies were omitted. Images were captured with an Olympus IX70 microscope equipped with a Leica DFC340 FX camera using LAS AF software (Leica).

Brightness and contrast adjustments consistent with image-manipulation policies were performed with either LAS AF, ImageJ, version 1.47a (National Institutes of Health, http://imagej.nih.gov/ij) or Adobe Photoshop CS6 (Adobe Systems) image-processing software.

## Results

**Fig. 1:**
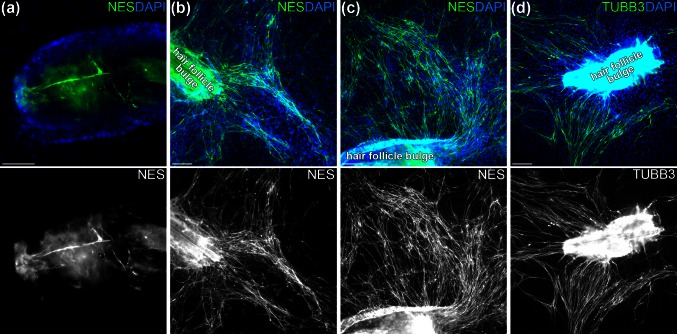
Nestin-positive and TUBB3-positive cells migrate from the hair follicle bulge. **a** Hair follicle bulge at the start of the culture, showing elongated nestin-positive (NES) cells (*green*) residing within the explant. **b**, **c** After 4 weeks in culture, numerous nestin-positive cells (*green*) are found growing in tracks extending from the bulge explant. **d** Hair follicle bulge explants after 4 four weeks of culture immunostain for TUBB3 in a pattern similar to that of nestin. Cell nuclei are stained *blue* with DAPI. *Scalebars* 100 μm (**a**) or 200 μm (**b**–**d**)

At the start of the culture, some cells residing within the hair follicle bulge stained positively for nestin, a marker commonly used to identify NCSCs (Fig. 1a). After 4 weeks, extensive outgrowth of nestin-positive bi- or tripolar cells migrating out of the explant was observed in each culture (Fig. 1b, c). Similar patterns were seen after immunostaining for TUBB3 (Fig. 1d). The nestin- and TUBB3-positive cells are positioned on top or in between of a matrix of flattened polyhedral cells. Based on previous immunophenotyping in similar culture experiments, these cells can be identified as pan-cytokeratin- and cytokeratin15-positive keratinocytes (results not shown).

**Fig. 2:**
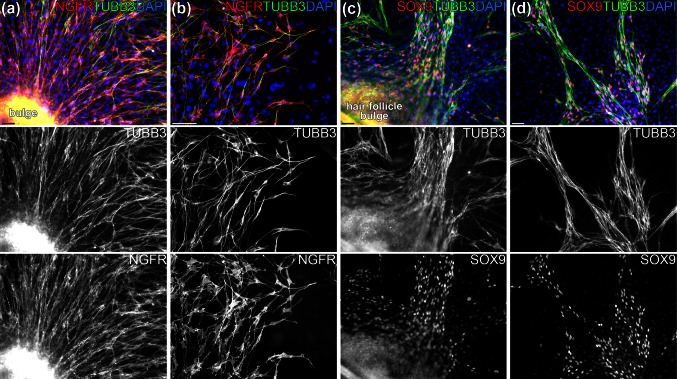
TUBB3-positive hair follicle bulge cells co-express NGFR and SOX9. **a**, **b** Double immunostaining for TUBB3 (*green*) and NGRF (*red*) showing that both proteins are expressed in the same subgroup of cells. **c**, **d** Double immunostaining for TUBB3 (*green*) and SOX9 (*red*). The strong co-expression of SOX9 indicates that the TUBB3-positive cells are of melanoglial, rather than neuronal, origin. Cell nuclei are stained *blue* with DAPI. *Scalebars* 50 μm

**Fig. 3:**
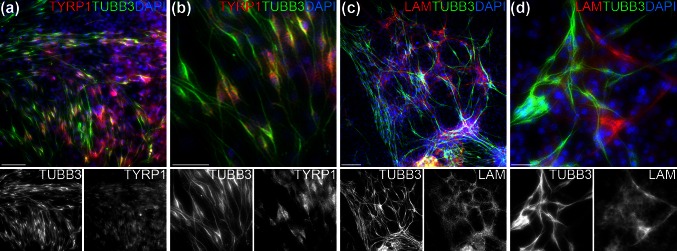
Melanocyte and glial marker expression of TUBB3-positive cells. **a** Hair follicle bulge explant after 4 weeks of culture immunostained for TUBB3 (*green*) and TYRP1 (*red*), showing weak expression of TYRP1 in the TUBB3-positive cells. **b** Higher magnification of the lower left area in **a**. **c**, **d** Double immunostaining for TUBB3 (*green*) and laminin (*red*) shows a subpopulation of laminin-positive cells largely growing along the same migration patterns of the TUBB3-positive cells. Cell nuclei are stained *blue* with DAPI. *Scalebars* 200 μm (**a**, **c**), 100 μm (**b**) or 20 μm (**d**)

To further establish the immunophenotype and identity these nestin- and TUBB3-positive cells, we performed double immunostaining for TUBB3 together with various markers of the early neural crest, Schwann cell and melanocyte lineages. All TUBB3-positive cells co-expressed nerve growth factor receptor (NGFR, also often named p75^NTR^) (Fig. 2a, b) and sex determining region Y-box 9 (SOX9) (Fig. 2c, d), indicating that these cells are of the melanoglial lineage. Double immunostaining for TUBB3 and tyrosinase-related protein 1 (TYRP1), a protein involved in melanin synthesis, revealed a high degree of co-expression of TUBB3 and (albeit weakly) TYRP1 (Fig. 3a, b). Double immunostaining for TUBB3 and laminin, an early Schwann cell marker, showed many TUBB3-positive cells and laminin-positive cells, but co-expression was never observed, suggesting two different cell populations. Remarkably, these cells seemed to migrate along the same tracks (Fig. 3c, d).

## Discussion

Based upon our previous results (Locher et al. 2013), we initially assumed that the TUBB3-positive cells migrating out of the hair follicle bulge explant would be melanocytes. Our results show, however, that the TUBB3-positive cells migrating out of the hair follicle bulge co-expressed nestin, NGFR and SOX9, indicative of a melanoglial identity, notwithstanding their TUBB3 expression which is generally thought to be limited to neuronal cells.

To investigate whether these cells were actually committed to either a melanocytic or glial cell fate, we performed additional double immunostainings for TUBB3 together with pertinent lineage markers for each respective cell type, i.e., TYRP1 for melanocytes and laminin for glial cells, resulting in diverse findings. TUBB3-positive cells showed weak TYRP1 co-expression, and the most plausible explanation is that these cells belong to a population of intermediate cells, which seem to show a tendency to the melanocytic commitment. Double immunostaining with antibodies against TUBB3 and laminin revealed a second population consisting of TUBB3-negative and laminin-positive cells, suggesting that these cells belong to the Schwann cell lineage.

The hair follicle bulge accommodates several different (stem) cell populations (Jaks et al. 2010). In addition, in the vibrissae this region is abundantly innervated by multiple sensory nerve endings (Takahashi-Iwanaga 2000; Maklad et al. 2004). Given the immunophenotype of the cells in our cultures, we hypothesize that both populations originate either from Schwann cells located in the proximal nerve stump or sensory nerve endings (Maklad et al. 2004; Woo et al. 2012; Kaucká and Adameyko 2014), or from melanocyte stem cells in the bulge (Dupin et al. 2003; Wong et al. 2006), both displaying an intermediate status between glial and melanocytic cell fate choice.

Recently, a nestin-positive cell population has been shown to reside between the basal membrane and the outer root sheath in the area just above the bulge, exhibiting long processes forming a crown around the whole hair follicle (Djian-Zaouche et al. 2012). These cells are closely associated with terminal nerve endings and are thought to be similar to nestin-positive type II terminal Schwann cells of the piloneural collar (Woo et al. 2012). Interestingly, the first paper reports that these nestin-positive cells co-express NGFR and that this nestin- and NGFR-positive cell population shares histological characteristics and immunological markers with the nestin-positive, multipotent stem cells from the bulge–isthmus region of hair follicles (Amoh et al. 2012). The authors assume that these cells all represent a single cell type originating from the piloneural collars. Therefore, it is conceivable that the TUBB3- and NGFR-positive cells found in our cultures originate from the same source, i.e., Schwann cells from the hair follicle sensory nerve endings, existing in an intermediate phase between a Schwann cell precursor and a melanocyte, as has been described previously (Adameyko and Lallemend 2010). It then seems plausible to assume that the TUBB3-negative but laminin-positive cells represent a population of more mature Schwann cells, although these different populations share a common predecessor. Whether the TUBB3-positive melanoglial cells are derived from this population by dedifferentiation or from distinct melanocyte stem cells located in the hair follicle bulge remains to be elucidated.

Interestingly, it has been reported that nestin- and NGFR-positive cells, identified as nerve-terminal-associated NCSCs and located around the hair follicle bulge, contribute cells to regenerating dermis following skin injury (Johnston et al. 2013). These nestin- and NGFR-positive cells are probably key to wound healing, for it has been shown that normal innervation is prerequisite for complete healing of injured skin (Kumar and Brockes 2012). As the nestin- and NGFR-positive cells in our study can be cultured easily, they could be used in future studies using wound healing models.

In this study, we have demonstrated that hair follicle bulge explants yield TUBB3-positive cells of melanoglial lineage, which sheds a new light on the different populations of (stem) cells that have been proposed to reside in the hair follicle bulge. In particular, the use of TUBB3 as a specific marker of neuronal identity should be considered with great caution, because cells of a melanoglial phenotype also do express TUBB3.
